# Targeting the MAPK Pathway in Cancer

**DOI:** 10.3390/ijms27010214

**Published:** 2025-12-24

**Authors:** Sultan F. Kadasah

**Affiliations:** Department of Biology, Faculty of Science, University of Bisha, P.O. Box 551, Bisha 61922, Saudi Arabia; sukadasah@ub.edu.sa

**Keywords:** cell, tumor microenvironment, mitogen-activated protein kinases, carcinogenesis, cell proliferation

## Abstract

The mitogen-activated protein kinase (MAPK) signaling cascade is fundamental in regulating cellular proliferation and differentiation, cell survival and cell death via apoptosis. Disruption of the MAPK signaling cascade at any point can lead to the evasion of apoptosis and unchecked cell growth and proliferation, leading to oncogenesis. This narrative review describes MAPK pathway dysregulation, its therapeutic targets, and resistance mechanisms. The therapeutic targeting of the MAPK pathway is complex due to the dual context-dependent roles of several kinases in the signaling cascade. Despite the therapeutic effectiveness of MAPK inhibitors, cancer cells develop chemoresistance that needs to be targeted via bypassing (c-Jun N-terminal kinases) JNK, protein kinase AKT and (mammalian target of rapamycin) mTOR signaling cascades, pairing MAPK inhibitors with multiple immune agents and targeting the MAPK pathway downstream of (extracellular signal-regulated kinase) ERK to prevent its reactivation mechanisms using combination therapies, downstream signaling regulators and (Proteolysis Targeting Chimeras) PROTACs. Additionally, MAPK-mediated regulation of ferroptosis is a novel oncological therapeutic targeting strategy for controlling tumor progression. The inhibition of the RAF/MAPK pathway results in alteration of several key regulators of ferroptosis, including SLCA11, GSH, GPX4 and NCO4A, hence affecting lipid cellular iron concentration and lipid peroxidation. Emerging therapies targeting the MAPK pathway should be designed considering crosstalk, compensatory signaling mechanism activation, the role of ferroptosis and the impact of the tumor microenvironment.

## 1. Introduction

Cancer is a leading cause of morbidity and mortality all around the world, accounting for almost 14.5% of total annual global mortality and 8.8% of disability-adjusted life years [1]. Aberrant molecular signaling in cancer cells is an essential component in the development of cancer [2]. Of all the molecular pathways regulating cell signaling and growth, the mitogen-activated protein kinase (MAPK) signaling cascade is fundamental in regulating cellular proliferation and differentiation, cell survival and cell death via apoptosis [3]. Moreover, this cascade is the cornerstone signaling pathway for mediating cellular response to stress [4]. Under physiological conditions, the MAPK signaling cascade is tightly regulated by certain extracellular stimuli such as cytokines, receptor tyrosine kinases and growth factors. These stimuli activate RAS (Rat Sarcoma), which leads to the translocation of extracellular signal-regulated kinase (ERK), Raf and MAPK/ERK (MEK), thus modulating genetic expression, cell cycle progression and their downstream effects [5].

Disruption of the MAPK/ERK signaling cascade at any point can lead to the evasion of apoptosis and unchecked cell growth and proliferation, leading to oncogenesis [6]. The dysregulation of the MAPK/ERK pathway and recurrent mutations are frequently observed across varied malignancies in humans [7]. According to Maik-Rachline, RAS mutations account for approximately 30% of mutations in all tumors [8]. In a large genomic study conducted by Sinkala et al., MAPK mutations accounted for almost 58% of mutations in all cancer types, with some of the cancer cells expressing exceptionally higher frequencies [9]. The literature has identified the occurrence of types of MAPK signaling dysregulation, including thyroid cancers, melanomas, colorectal cancers and several others, which makes it an attractive therapeutic target for treating oncological diseases [7]. Targeting the MAPK pathway, an essential mediator of the cell cycle, results in impaired cell growth and apoptosis in tumor cells [10]. Several cancer treatment strategies have been developed to target the MAPK pathway at various stages of clinical trials, with variable results [10].

However, the complexity of MAPK signaling in the form of complicated feedback loops, crosstalk with other signaling pathways and resistance to targeted therapy via the activation of bypass signaling pathways might affect the therapeutic success of targeted therapies [11]. Overall, the key role of the MAPK pathway in tumorigenesis necessitates studying the role of MAPK as an essential therapeutic target in cancer. This narrative review aimed to describe the molecular underpinnings of MAPK pathway dysregulation, identify currently available therapeutic targets, determine resistance mechanisms, and underscore future research required for improving patient outcomes.

## 2. Overview of the MAPK Pathway in Cancer

The MAPK signaling cascade comprises sequential three to five layers of kinases; however, the number of these layers can vary in different types of cells [12]. It includes, as a basic core unit, the first three layers available in all types of cells, including MAPK kinase kinase (MAP4K), MAPK kinase (MAP3K), MAPK (MAPKK), and MAPK-activated protein kinases (MAPKAPK), with the last two layers comprising MAPK and MAPK-activated protein kinases (MAPKAPK) [12]. The MAPK signaling cascade is broadly classified into four categories based on the structural components: ERK1/2, c-Jun N-terminal kinase (JNK), p38 MAPK and ERK5 [12]. Extracellular stimulus triggers the activation of MAPKKK via phosphorylation, in turn phosphorylating MAPKKs [13]. Resultantly, the tripeptide motif (Thr-X-Tyr) in the activation loop of MAP kinase is activated, which mediates downstream signaling. In some cells, this three-tier MAPK cascade is supplemented with regulatory layers of the carboxyl terminal, which is involved in nuclear translocation and transcription [13].

## 3. Canonical MAPK/ERK Cascade

**Figure 1:**
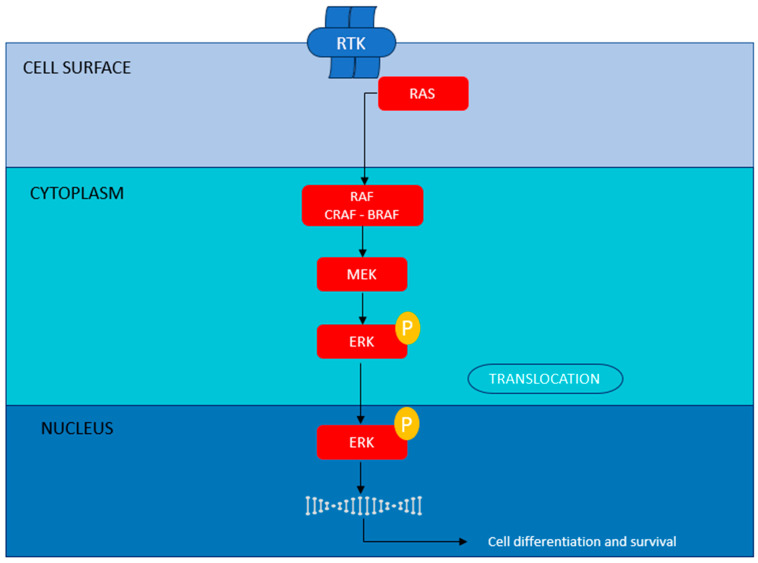
The canonical MAPK cascade.

Of the discussed MAPK signaling cascades, MAPK/ERK signaling is most frequently discussed in cancer due to its effective role in cell proliferation and differentiation [10]. The phosphorylation cascade begins with RAS (Figure 1), an essential upstream protein in the RAF/MEK/ERK pathway. The binding of ligands with receptor tyrosine kinases (RTKs) results in the loading of RAS with GTP and the recruitment of adapter proteins such as GRB2/SOS [14]. This results in the activation of RAF kinases (ARAF, BRAF, CRAF), leading to the phosphorylation and activation of dual-specificity kinases MEK1/2, followed by the phosphorylation of ERK1/2 [10]. The phosphorylated ERK1/2 is translocated into the nucleus, where several transcription factors are activated, including ELK1, c-Fos and c-Myc. These factors are pivotal in regulating the cell cycle, cell metabolism and cell survival [10,15,16]. The specificity of outcomes in the whole signaling cascade is largely determined by the amplitude of the stimulatory signal, the duration of stimulation, the type of subcellular localization, and the types of scaffold proteins activated in the pathway [17].

## 4. Oncogenic Mutations in the MAPK/ERK Cascade

The well-controlled MAPK/ERK cascade experiences unchecked overactivation in various cancers due to the activation of mutations in components of the MAPK cascade, which drives constitutive signaling, rendering it an always-on pathway (Table 1). This drives the uncontrolled proliferation of cells, with angiogenesis, the evasion of apoptosis and metastasis occurring [7]. RAS and Raf mutations are encountered most frequently in various cancers, with RAS mutations accounting for almost 30% of all cancer types [18]. Among RAS mutations, the most frequently found oncogenic driver is KRAS mutations, observed in almost 97.7% of pancreatic ductal adenocarcinoma, 44.7% of colorectal carcinoma, and 30.9% of lung carcinoma cases [19]. NRAS mutations are common in melanomas and hematological malignancies, whereas HRAS mutations are frequently observed in head and neck squamous cancers (4.7%), as well as urothelial carcinomas (5.9%) [19]. This varied distribution of various oncogenic mutations in MAPK signaling in different cancers makes it an attractive therapeutic target; however, some of these mutations are challenging due to their undruggable structures [20]. The diversity of the mutations highlights the need for precision therapeutic approaches tailored according to mutation type and tumor context.

## 5. Dysregulation Beyond Mutations

- JNK (c-Jun N-terminal kinase) Pathway—A Double-Edged Sword and Therapeutic Opportunities

Besides the MAPK/RAS/RAF/ERK pathway, the c-Jun N-terminal kinase (JNK) pathway is another type of MAPK signaling cascade comprising serine/threonine kinases, which are activated by stress, reactive oxygen species, cytokine storm and genotoxic stress [28]. Transcription factors such as c-Jun, ATF2, p53, and c-Myc and apoptotic proteins such as BCL-2 and cytoskeletal regulators are resultantly phosphorylated, thus serving as mediators of cellular stress response and cell-fate decisions [28]. Unlike the MPK/ERK cascade, which undergoes point mutations, JNK signaling is dysregulated by isoform imbalance, shifts in scaffold proteins and disturbance in upstream stimulus. This renders JNK a functional therapeutic target in various cancers [29].

## 6. Dual Roles: Apoptosis Promoter vs. Tumor Facilitator

The biology of JNK in cancers is context-dependent, thus playing a dual role in cancer. In some cancers, the activation of JNK promotes apoptosis, while under other conditions, it leads to increased tumor cell proliferation and increased tumor invasion, survival and therapy resistance [30]. The outcomes of JNK activation largely depend upon the amplitude and duration of JNK activation, the crosstalk of JNK with other molecular cascades (PI3K and p38/ERK), and the relative activity of JNK isoforms (JNK1 vs. JNK2 vs. JNK3) [30]. This tissue- and stimulus-dependent effect of JNK makes it an essential therapeutic target in oncology (Table 2).

## 7. p38 MAPK Pathway in Oncogenesis

The p38 pathway, also known as stress-associated protein kinases (SAPKs), is usually activated via genotoxic or environmental stress [38]. There are four types of p38 MAPKs, namely p38α and p38β (MAPK11), p38γ (MAPK12) and p38δ (MAPK13), coded by MAPK14, MAPK11, MAPK12 and MAPK13, respectively [39,40]. The activation of p38 signaling occurs via the dual phosphorylation of Thr and Tyr on the Thr–Gly–Tyr motif, located on kinase subdomain VIII [41]. The activation of the p38 MAPK pathway under conditions of environmental stress and toxins is mediated through the phosphorylation of threonine 180 and tyrosine 182 residues via MAP3Ks and MKK3/6 [38,42,43,44,45]. Moreover, several non-canonical pathways might also result in the activation of p38 signaling in T-lymphocytes and myocytes [46]. The p38 signaling pathway regulates cellular proliferation, differentiation and survival, apoptosis and stress response [40,46], which are necessary mechanisms determining cellular oncogenesis.

## 8. Dual Role of the p38 Signaling Cascade in Oncogenesis

The role of the p38 pathway in cancers is complex, with the literature sometimes presenting it as a tumor suppressor pathway, on one hand, or a tumor promoter pathway, on the other hand. p38α ablation results in the modulation of Epo expression, which increases the proliferation of hematopoietic progenitor cells [47]. Similarly, p38α-deficient mice were previously found to be susceptible to developing lung and liver cancers [48,49]. The downregulation of cell cycle proteins mediated via p38-phosphorylated RB also inhibits cell proliferation [50]. In contrast, the deletion of the p38α gene in a breast cancer model resulted in decreased tumor volume [51]. The dual role of p38 signaling in oncogenesis is detailed in Table 3.

## 9. Oncological Targeting of MAPK Pathway

Many researchers are struggling to develop therapeutic targets for enabling the JNK pathway to halt cancer cell growth. In pancreatic cancer, several JNK inhibitors have been developed to date, with variable isoform selectivity. SP600125, a reversible ATP-competitive pan JNK-inhibitor, has shown great effectiveness in reducing pancreatic cancer cell growth [31,59]. SP600125, when combined with radiotherapy, has decreased the resistance of metastatic tetraploid cells by inhibiting the JNK pathway [60]. Bentamapimod (AS602801), an ATP-competitive inhibitor pan-JNK inhibitor, is effective at reducing cell survival and tumorigenesis in pancreatic cancers, glioblastomas and ovarian cancers [61,62,63]. In endometrial cancers, Bentamapimod induced the regression of endometrial lesions by inhibiting JNK signaling, resulting in the inhibition of cytokine secretion and progesterone resistance [64]. Lichocholane, a JNK1-specific inhibitor, inhibits cancer cell survival by competing with the JIP1 scaffolding protein in binding with JNK1 [65]. The JNK-inhibitor-IX, a JNK2-specific ATP-competitive inhibitor, has shown superior effectiveness in halting the progress of PANC-1, the most common cell line in pancreatic cancers that is resistant to JNK inhibition [66,67]. Several BRAF and MEK inhibitors, such as vemurafinib and dabrafenib, are used in various cancers, including melanomas, thyroid cancers, lung cancers, and many others [68]. Sorafenib also targets the RAF/MEK/ERK pathway to limit the progression of hepatocellular carcinoma [69]. Ulixertinib, an ERK inhibitor, has been used in treating non-small-cell lung carcinoma, colorectal carcinoma and melanoma [70]. It has also shown great effectiveness in pediatric low-grade glioma models when used as a monotherapy, as well as an adjunct to BH3-mimetics, chemotherapy or MEK inhibitors [71]. For the combination of dabrafenib with trametinib, BRAF and MEK inhibitors have shown improved progression-free survival and overall survival, respectively, in several trials of patients with BRAF-mutant solid tumors [72]. Sotorasib, a KRAS^G12C^-irreversible inhibitor, achieved significantly greater median progression-free survival in patients with advanced non-small-cell lung cancer (NSCLC) compared with docetaxel, i.e., 5.6 versus 4.7 months (*p* < 0.001) [73]. Similarly, another KRAS^G12C^-irreversible inhibitor, Adagrasib, has been proven to be effective in treating KRAS^G12C^-mutated NSCLC in patients refractory to platinum-based chemotherapy and anti-programmed death ligand 1 therapy [74]. Selumitinib, a MEK1/2 inhibitor, has shown clinical and radiological improvement in patients suffering from inoperable symptomatic plexiform neurofibroma with an effective safety profile [75]. Tunlametinib, another MEK inhibitor, resulted in significant antitumor activity in patients with inoperable, stage III/4 NRAS-mutant melanomas with prior exposure to immunotherapy [76]. Additionally, several combinational–sequential strategies have been proposed in the literature for improving the clinical efficacy of MAPK targeting, while overcoming drug resistance [77]. Wang et al. proposed that sequencing only two doses of anti-PDL-1 or anti-CTLA-4 agents before initiating MAPK inhibitors in melanoma improves both their antitumor activity and the efficacy of clinical therapy [77].

Collectively, this demonstrates how several clinically approved and investigational drugs can provide meaningful antitumor activity via targeting the MAPK oncogenic axis in diverse malignancies.

## 10. MAPK Targeting and Drug Resistance

**Figure 2:**
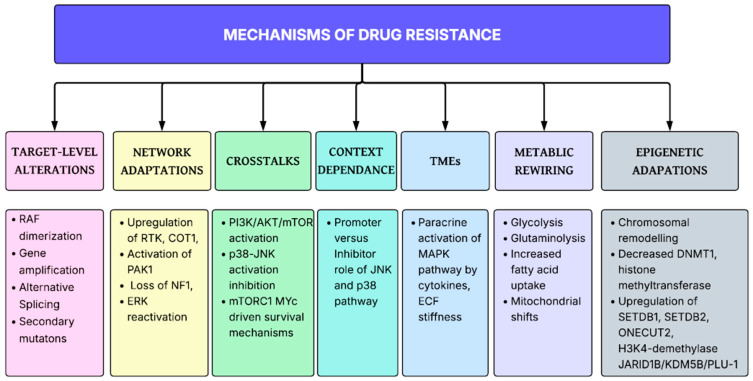
The drug resistance mechanisms in the MAPK signaling cascade.

Despite the therapeutic effectiveness of agents targeting the MAPK signaling cascades in cancers, it is evident from the literature that several resistance mechanisms have emerged that limit the durability of the therapeutic effects of these drugs. Sturm et al. proposed negative feedback amplification as a resistance mechanism against MEK inhibitors that sustains ERK signaling [78]. Combining MEK inhibitors with RAF inhibitors overcomes the negative feedback amplification of MEK; therefore, this combination is now used as a standard therapy in treating malignant melanoma [79,80]. Unfortunately, several other escape mechanisms have been developed against RAF inhibitors that reactivate the ERK pathways, including RAF protein dimerization, RAF amplification and splicing mutations [81]. Additionally, the activation of alternative pathways has resulted in resistance against combination therapies combining MEK inhibitors with RAF inhibitors [82]. According to one in vitro study, targeting the p38 pathway using inhibitors results in the increased proliferation of pancreatic cancer cells via the activation of the JNK pathway [83]. Zhong et al. proposed inhibiting pancreatic cancer cell growth via p38 MAPK activation, which inhibits the JNK pathway [83]. Ning et al. also proposed carrying out the phosphorylation activation of the JNK p38 pathway via the inhibition of the p38 pathway and vice versa [84]. Morphine, at low doses, can promote pancreatic cancer progression via the activation of the JNK pathway secondary to p38 inhibition, whereas at high doses, it activates p38, leading to the suppression of the JNK pathway and, thus, of cancer growth [84]. This highlights that the compensatory activation of parallel pathways in cancers may limit the therapeutic success of isolated inhibitors. Additionally, the feedback reactivation of receptor tyrosine kinases [85], epigenetic modulation [86] and the tumor microenvironment [87] played important roles in mediating therapeutic resistance, as shown in Figure 2.

## 11. Targeting Drug Resistance in MAPK Tumor Therapy

The clinical effectiveness of MAPK-targeted tumor therapy is limited by several resistance mechanisms that need to be considered when initiating MAPK-targeted antitumor therapy in patients with oncological diseases. Studies in the recent literature have basically focused on three mechanistic approaches: combination therapies for bypassing several molecular signaling points, the pairing of MAPK inhibitors with multiple immune agents and targeting the MAPK pathway downstream of ERK to prevent its reactivation [88,89].

The therapy combining axitinib with pembrolizumab and avelumab has led to improved overall survival, progression-free survival, and response rates in renal cell carcinoma [90,91]. Similarly, in EGFR-mutant metastatic NSLC, combining erlotinib with ramucirumab resulted in improved progression-free survival in a RELAY Phase III trial [92]. In hepatocellular carcinoma, using combination immunotherapy combining MAPK inhibitors, i.e., atezolizumab and bevacizumab, with sorafenib resulted in improved overall survival and progression-free survival compared to monotherapy [93]. In a phase III clinical trial of advanced renal cell carcinoma, CheckMate-9ER, overall response and the survival rate were higher in patients who received nivolumab plus cabozantinib compared to sunitinib alone [94]. However, not all combination therapies were effective: Kelley et al. observed no significant difference in the overall survival rate in patients with melanomas when treated with sorafenib alone or combination therapy combining cabozantinib with atezolizumab [95]. This disagreement highlights the need for future clinical trials. Nelfinavir, an HIV-1 protease inhibitor, is an essential salvage therapy for treating non-mutational drug tolerance developed in BRAF- and NRAS-mutant melanomas during the therapy phase due to a PAX-3-mediated increased expression of the MITF gene [96]. In ovarian carcinomas, an alpha1-antiagonist, Nafttopidil, has shown greater efficacy in overcoming resistance to MEK inhibitors via the activation of the JNK signaling pathway and stimulation of BH3-only protein expression [97].

Kinases downstream of ERK, including p90 ribosomal S6 kinase (RSK), which is involved in transcription and translation mediated via the Y-box binding protein 1, also result in improved tumor cell survival despite treatment with chemotherapy [98]. Targeting YB-1 using RSK inhibitors re-sensitizes vemurafinib-resistant melanoma cells to BRAF inhibitors [99]. A novel RSK inhibitor, PMD-026, has shown high specificity in triple-negative breast carcinoma [100]. In several phase 1 clinical trials of metastatic triple-negative breast carcinoma, PMD-026 has demonstrated an effective safety profile compared with inhibitors using the MAPK pathway [100,101]. RSK inhibitors have also been tested in prostate carcinoma in in vitro and in vivo studies [102]. RSK inhibitors are known to improve the responses of endogenous and adaptive T cells directed against melanocytic differentiation antigens, thus improving the responsiveness of melanoma cells to immunotherapy [103]. The combination of CDK4/6 inhibitors with MAPK inhibitors is another therapeutic option for targeting cancers resistant to MAPK inhibitors alone. This combinatorial therapy works by modulating the cell cycle and altering the transcriptional expression of CDK6 and AP-1 transcription factors [104]. The combined therapy of RAF inhibitors with abemaciclib, a CDK4/6 inhibitor, resulted in the regression of tumors in a KRAS-, NRAS- and BRAF-mutant xenograft model [105]. It induces apoptosis in BRAF-inhibitor-resistant cells and stimulates cell cycle arrest in non-resistant cells [106]. Similarly, combination therapy combining trametinib with palbociclib resulted in NRAS-mutant melanoma models [107]. In neuroblastoma models, combination therapy combining binimetinib with ribociclib resulted in impaired tumor growth [108].

Recent advancements in targeting resistance to anti-cancer therapy have resulted in the development of proteolysis-targeting chimeras (PROTACs). In BRAF-mutant cancers, BRAF-PROTACs were developed by Cullgen as compounds 12 and 23 by linking BI882370, a pan-RAF inhibitor, and vemurafinib to the CRBN thalidomide ligand [109]. Similarly, SJF-0628 results in the degradation of BRAF-mutant cancers in a dose-dependent manner within four hours with no ERK phosphorylation for up to 72 h and only 30% recovery of BRAF activity after washout within 24 h, highlighting the extended catalytic activity of BRAF-PROTAC treatment [110]. P4b, another CRBN-recruiting PROTAC developed by combining dabrafenib with BI882370, resulted in reduced cellular proliferation in vemurafinib-resistant cells [111]. In KRAS^G12D^-mutant pancreatic cancer, a CRBN-based PROTAC, ZJK-807, has shown effectiveness in targeting chemoresistance to MRTX1133 by uniquely modulating the signaling mechanism of TNF and eukaryotic ribosomal biogenesis, thus suppressing the growth of chemoresistant cells [112].

Although all these described inhibitors have proved effective in targeting cancers via the MAPK signaling cascade, it should be noted that these inhibitors are at different stages of clinical development. Most of the RAF and MEKs, including vemurafenib, dabrafenib, sorafenib and trametinib, are FDA-approved, while the novel ERK inhibitors and PROTACS are either in pre-clinical or early phases of development, which needs to be considered while contextualizing clinical translational relevance.

## 12. Role of MAPK Targeting with Ferroptosis Regulation in Oncology

**Figure 3:**
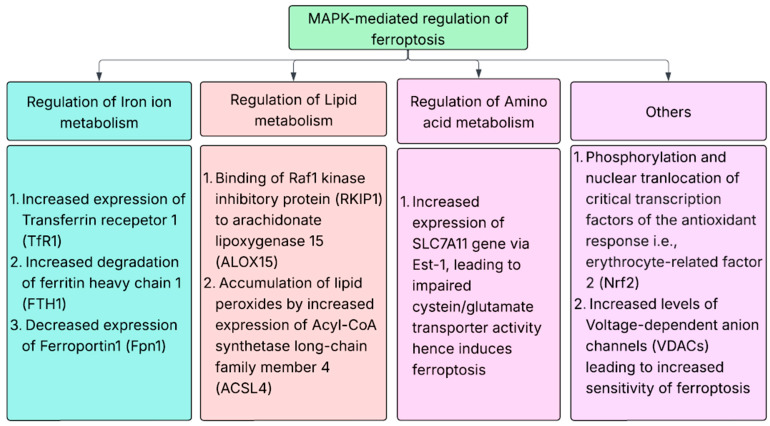
MAPK modulation of ferroptosis.

Ferroptosis, a form of programmed cell death that occurs due to increased levels of iron and lipid peroxidation of fatty acids in cell membranes, is highly modulated via the MAPK signaling cascade [113]. Increased cellular levels of lipid peroxidation and reactive oxygen species induce ferroptosis via the MAPK signaling cascade [114]. The increased activation of erastin-mediated stimulation of ferroptosis has been observed in RAS-mutated cancer cell lines [115]. The molecular mechanisms through which ferroptosis is modulated via MAPK signaling include regulation of iron-ion homeostasis, the regulation of lipid and amino acid metabolism and alterations in the expression levels of various factors, including nuclear factor erythrocyte-related factor 2 (Nrf2) and voltage-dependent anion channels (VDACs) present between the mitochondria and cytoplasm [116,117,118]. Riaz et al. also identified MAPK1 as an essential ferroptosis-related gene in triple-negative breast cancers due to its role in cellular proliferation and susceptibility to ferroptosis. Conversely, the inhibition of the MAPK pathway results in improved ferroptosis by disrupting cellular signaling, especially in cancers with NF2 or RAS mutation [119]. A summary of the molecular mechanism through which the MAPK signaling cascade modulates ferroptosis is provided in Figure 3.

## 13. MAPK Modulation of Ferroptosis—Its Therapeutic Role in Oncology

The RAS synthetic lethal screen has described the active role of the RAS oncogene in modulating ferroptosis via erastin and RSL3 [120,121]. The inhibition of the RAF/MAPK pathway has been shown to reverse cytotoxicity-mediated by erastin or RSL3 in RAS-mutant tumor cells via the modulation of iron-metabolism gene expression [118]. Mutation in the epidermal growth factor receptor (EGFR) results in increased ferroptosis sensitivity in mammary epithelial cells and non-small-cell lung cancers [122]. The evasion of ferroptosis in RAS-mutant cells has established its vulnerability in lung cancer [123].

Several studies have described the regulation of ferroptosis in cancer cells to limit their growth [116,124,125]. In KRAS-mutated colorectal carcinoma, Cetuximab, an anti-EGFR antibody, leads to the depletion of Glutathione (GSH) when combined with β-Elemene by inducing the iron-dependent accumulation of reactive oxygen species [125]. Similarly, in breast cancer, the therapeutic induction of ferroptosis is possible by blocking the transport of iron via combining Siramesine with Lapatinib, an inhibitor of EGFR and HER2 [126,127]. For adenocarcinoma in the lung with the EGFR-mutant strain, the induction of ferroptosis halts the progression of cancer upon the administration of Vorinostat, a histone deacetylase inhibitor that mediates decreased SLC7A11 expression [124].

JNK activators have also highlighted cancer cells’ positive sensitization towards ferroptosis. SP600125, a JNK-IN-8 activator, promotes the nuclear translocation of RB1CC1 (RB1-inducible coiled-coil 1) and stimulates ferroptosis sensitivity [128]. RB1CC1, an essential factor for the development of autophagosomes, modulates cellular proliferation via the positive regulation of lipid ROS [128]. The Ser537 phosphorylation-dependent nuclear translocation of RB1CC1 initiates transcriptional reprogramming, improving ferroptosis sensitization. Nuclear RB1CC1 increases the number of histone modifications (H4K12Ac) present at ferroptosis-linked enhancers by recruiting elongator acetyltransferase complex subunit 3 (ELP3) through forkhead box (FOX)-binding motifs, thus stimulating ferroptosis-associated genes such as CHCHD3 to improve mitochondrial functioning, as shown in lung cancer models [129]. Despite this positive role of JNK activators in sensitizing cancer cells towards ferroptosis, the dual role of JNK activators needs to be considered when prescribing therapy, as these activators have shown proinflammatory effects in healthy tissues [128].

Anisomycin, a p38MAPK activator, promotes the phosphorylation of H3S10, which activates the NCOA4 gene. NCOA4, a ferroptosis stimulator gene, is involved in the recruitment of FTH1 to autophagosomes, thus driving its lysosomal degradation. This results in an increased iron pool mediated via ferritinophagy, which stimulates cell death in hepatic cancer cells due to altered iron levels, stimulating lipid peroxidation via the Fenton reaction. Simultaneously, anisomycin negatively regulates solute carrier family 7 member 11 (SLC7A11) via the p38-MAPK pathway through non-GPX4 inactivation [130].

In KRAS-mutant cancers with G12Ci-resistant cells, SOX2, SLC7A11 and SLC40A1 are downregulated. Treating KRAS-mutant cancers with G12Ci results in the feedback activation of the MAK pathway, which modulates ferroptosis in cancer cells, thus suggesting that the MAPK-SOX2 axis is an essential target for the modulation of ferroptosis in G12Ci-resistant tumors via the regulation of intracellular GSH synthesis [131].

## 14. Conclusions

The ERK, JNK and p38 pathways are essential molecular signaling cascades that regulate the oncogenic transformation of cells throughout the body. Any dysregulation in these signaling cascades as a result of genetic mutations, aberrant kinase activity or disturbed feedback activation results in tumorigenesis and malignancies. Although strategies for the isolated targeting of each pathway up to the point of clinical translation have been developed, the therapeutic targeting of the MAPK pathway is complex due to the dual context-dependent roles of several kinases in the signaling cascade. Despite the therapeutic effectiveness of MAPK inhibitors, cancer cells develop chemoresistance that needs to be targeted via bypassing several molecular signaling points, pairing MAPK inhibitors with multiple immune agents and targeting the MAPK pathway downstream of ERK to prevent its reactivation mechanisms using combination therapies, downstream signaling regulators and PROTACs. Additionally, the MAPK-mediated regulation of ferroptosis is a novel oncological therapeutic targeting strategy for controlling tumor progression. The emerging therapies targeting the MAPK pathway should be designed considering these inter-pathway interactions, compensatory signaling mechanism activation, and the impact of the tumor microenvironment.

## Figures and Tables

**Table 1:** MAPK mutations in various cancer types.

Cancer Type	MAPK Mutation
Pancreatic ductal adenocarcinoma [19,21]	KRAS, BRAF
Colorectal carcinoma [22,23]	KRAS (G12D, G12C, G12S, G13D, Q61R, Q61H, Q61L) NRAS (G13A and Q61H), BRAF (V600)
Breast carcinoma [24,25]	KRAS, NRAS, MKP1, MKP2
Lung carcinoma [26,27]	BRAF
Biliary carcinoma	MKP1, MKP2, JNK activity, MKK4

**Table 2:** The dual role of the JNK cascade in oncogenesis.

Cancer Type	Mechanism and Therapeutic Implications	Dual Role	References
Pancreatic cancer	JNK2 inhibition increases invasion JNK1 inhibition leads to tumor growth suppression Need for isoform-selective therapeutic targeting strategies	Pro-apoptotic and Pro-tumorigenic	[31]
Bladder cancer	JNK inhibition decreases the cancer-associated fibroblast-mediated expression of thymic stromal lymphopoietin (TSLP) required for creating an immunosuppressive microenvironment JNK inhibition with anti-PD-1 treatment is effective against bladder cancer	Pro-tumorigenic	[32]
Colorectal carcinoma	LINC02257/JNK axis leads to colorectal liver metastasis	Pro-tumorigenic	[33]
Glioma	Pro-apoptotic in glioma cells, pro-proliferative in vascular smooth muscle cells	Pro-apoptotic and Pro-tumorigenic	[34,35]
Vestibular schwannoma	Inhibits the apoptosis of cancer cells by limiting ROS accumulation	Pro-tumorigenic	[36]
Lymphoma	Inhibits the apoptosis of cancer cells by limiting ROS accumulation; combination therapy combining bortezomib with JNK inhibitors is required	Pro-tumorigenic	[37]

**Table 3:** The dual role of the p38 signaling cascade in oncogenesis.

Type of Cancer	p38 Isoform	Evidence	Animal Model/Cell Used	Reference
Breast cancer	p38α	Deletion leads to altered DNA damage response	Mice	[52]
	p38δ	Deletion leads to decreased tumor volume	Mice	[51]
Lung cancers	-	Increased p38 kinase activation	Human lung cancer cell	[53]
Head and neck cancers	-	Hyperactivated p38 in tissue samples	Human head and neck cancer cell	[54]
Colon cancers	p38γ	Increased expression leading to increased proliferation	NA	[55]
	p38α	Increased expression leading to increased proliferation	Mice	[56]
Liver cancers	p38γ	Deletion or inhibition reduces the formation of liver tumors induced by chemicals	Mice	[57]
Bladder carcinoma	p38α	Inhibition leads to the reduced invasion of cancer cells by diminishing MMP-2/9 activities	Human bladder cancer cells	[58]

## Data Availability

No new data were created or analyzed in this study. Data sharing is not applicable to this article.
